# A collaboratively produced model of service design for children and young people with common mental health problems

**DOI:** 10.1186/s12913-024-10562-7

**Published:** 2024-01-24

**Authors:** Steven Pryjmachuk, Susan Kirk, Claire Fraser, Nicola Evans, Rhiannon Lane, Jodie Crooks, Rose McGowan, Georgia Naughton, Liz Neill, Elizabeth Camacho, Peter Bower, Penny Bee, Tim McDougall

**Affiliations:** 1 School of Health Sciences, The University of Manchester and Manchester Academic Health Science Centre (MAHSC), Jean McFarlane Building, Oxford Road, Manchester, M13 9PL UK; 2 Manchester Mental Health NHS Foundation Trust, Manchester, UK; 3 School of Healthcare Studies, Cardiff University, Cardiff, UK; 4 The McPin Foundation, London, UK; 5 Common Room North, Leeds, UK; 6 University of Liverpool, Liverpool, UK; 7 Lancashire and South Cumbria NHS Foundation Trust, Preston, UK

**Keywords:** Children, Young people, Mental health services, Organisational case studies, Evidence synthesis, Service design, Service delivery

## Abstract

**Background:**

Little is known about the effectiveness of, and implementation complexities associated with, service delivery models for children and young people (CYP) experiencing ‘common’ mental health problems such as anxiety, depression, behavioural difficulties and self-harm. This paper outlines how a model for high-quality service design for this population group was developed by identifying available services, their effectiveness, cost-effectiveness and acceptability, and the barriers and enablers to access.

**Methods:**

Sequential, mixed-methods design, combining evidence syntheses (scoping and integrative reviews of the international literature) with primary research (a collective case study in England and Wales). Data from these two elements were collaboratively synthesised in a subsequent model-building phase.

**Results:**

The scoping review yielded a service model typology. The integrative review found effectiveness evidence only for four models: collaborative care (the only service model to also have cost-effectiveness evidence), outreach approaches, brief intervention services and an organisational framework called ‘Availability, Responsiveness and Continuity’. No service model seemed more acceptable than others. Three case study themes were identified: pathways to support; service engagement; and learning and understanding. The model-building phase identified rapid access, learning self-care skills, individualised support, clear information, compassionate and competent staff and aftercare planning as core characteristics of high-quality services. These characteristics were underpinned by four organisational qualities: values that respect confidentiality; engagement and involvement; collaborative relationships; and a learning culture.

**Conclusions:**

A consistent organisational evidence-base for service design and delivery in CYP’s mental health spanning many years appears to have had little impact on service provision in England and Wales. Rather than impose – often inflexible and untested – specific local or national models or frameworks, those commissioning, designing and delivering mental health services for CYP should (re)focus on already known, fundamental components necessary for high-quality services. These fundamental components have been integrated into a collaboratively produced general model of service design for CYP with common mental health problems. While this general model is primarily focused on British service provision, it is broad enough to have utility for international audiences.

**Supplementary Information:**

The online version contains supplementary material available at 10.1186/s12913-024-10562-7.

## Background

The mental health of children and young people (CYP) has been a major public health concern for at least a decade, both in the United Kingdom (UK) [1–5] and internationally [6]. Recent estimates suggest around one in six 7–16 year olds and one in four 17–19 year olds in England may be experiencing significant mental health difficulties, including the likes of anxiety, depression, self-harm, behavioural difficulties and eating disorders [7].

As in adult service provision, the mental health conditions affecting CYP can be split into two broad categories: emotional and behavioural problems like anxiety and depression that affect many CYP; and less common mental health problems like schizophrenia. In the UK (particularly in adult services), the former are frequently referred to as ‘common’ mental health problems and the latter ‘serious’ mental health problems. This distinction is not always helpful, however, since common mental health problems often lead to serious difficulties for CYP and families. A more considerate distinction may thus be ‘common’ and ‘less common’ mental health problems.

The four-tiers model has dominated UK service provision in children’s mental health for at least two decades, emerging from a seminal government-backed report published in 1995 [8]. Tier 1 often involves non-mental health professionals and is focused on mental health promotion, mental ill-health prevention and screening in universal children’s services such as schools and primary care. Tiers 2 and 3 tend to be outpatient services, with Tier 3 services having more professionals involved and higher levels of input than Tier 2 services. Tier 4 services are very specialised services which usually means inpatient care. The model expected CYP to enter at the lower tier and progress to a higher tier only if support and treatment did not work at a lower tier.

Over the years, it has become increasingly clear that the four-tier model has not met the needs of most CYP experiencing mental health problems. Numerous reports and reviews [1, 3, 9–11] have consistently described UK children’s mental health services as fragmented, uncoordinated, variable, inaccessible and lacking an evidence-base. In response to these criticisms, there have been attempts to transform services using initiatives such as the Choice and Partnership Approach (CAPA) [12], CYP-IAPT (an analogue of the adult *Improving Access to Psychological Therapies* initiative for children and young people) [13] and ‘THRIVE’ [14, 15]. CAPA, developed in the early 2000s, was an initiative designed to improve service effectiveness and the management of service demand and capacity. The ‘choice’ in CAPA refers to giving CYP and families choice (in appointments, treatment options, and whether they engage further, for example); ‘partnership’ is what CAPA calls treatment or intervention, mainly because it is predicated on shared decision-making. CYP-IAPT was a government-supported initiative of the 2010s. Like its adult IAPT counterpart, CYP-IAPT aimed to improve the availability of, and access to, evidence-based psychological therapies. Unlike its adult counterpart, CYP-IAPT did not involve the recruitment and development of new types of workers; instead, it championed the training of existing staff in evidence-based therapies such as cognitive-behavioural therapy (CBT), parenting and interpersonal therapy (IPT). A more recent development is THRIVE (not an acronym although stylised in capitals). THRIVE is a framework for creating coherent and resource-efficient ‘communities’ of mental health that focuses on clarity around *need* rather than structures or interventions to meet such needs. THRIVE has been mooted as an alternative to the tiers model [14] with the four tiers being replaced by five (increasingly complex) levels of need: thriving, getting advice, getting help, getting risk support, and getting more help.

Little is known about the effectiveness of these initiatives nor the effectiveness of mental health service models for CYP in general. Moreover, the disparate factors associated with accessing and navigating services for CYP experiencing common mental health problems have not to date been synthesised into a coherent model of effective and acceptable service provision.

This paper outlines how a co-produced, evidence-based, general model of service design for CYP experiencing common mental health problems was developed via a mixed-methods study that encompassed an evidence synthesis, primary research and a model building phase. The study was informally named ‘Blueprint’ by the study’s young advisors. This paper focuses on the model building phase though some brief detail on the evidence synthesis and primary research phases is necessary for context.

## Methods

The study’s overarching aim was to develop a model of high-quality service design for CYP experiencing common mental health problems by identifying available services, the barriers and enablers to access, and the effectiveness (including cost-effectiveness) and acceptability of those services. Using a sequential, mixed-methods design, we combined evidence synthesis with primary research, subsequently synthesising the data from these two study elements into a general model of high-quality service design for this population group.

### Evidence synthesis phase

The evidence synthesis phase comprised a scoping review in which various service delivery models were identified and categorised and an integrative review exploring the evidence for the various models identified in the scoping review. A single literature search underpinned both reviews. Relevant international bibliographic databases and resources were searched in May 2019 using appropriate search terms. Additional documents (obtained from screening reference lists of relevant literature reviews, citations in included documents and the research team’s networks) continued to be added to the literature pool until the end of 2020.

A PICOS (Population, Intervention, Comparator, Outcomes and Study Design) formulation [16] was used to frame the inclusion criteria for the reviews (Table 1).

**Table 1:** PICOS inclusion criteria

	Scoping	Integrative
P: Population	The service’s key target group is those aged under 18 ‘Common mental health problems’ agreed by the study advisory group to be: anxiety and related disorders; depression; self-harm; PTSD; emerging personality disorder; adjustment disorder; ADHD/ADD; conduct disorder; oppositional defiant disorder; substance misuse disorders; ‘at risk of psychosis’ Note: the funding body’s call for research proposals specifically excluded psychosis, eating disorders and autism spectrum disorders because other research projects in their portfolio were investigating these conditions	
I: Intervention	Any service provided for CYP experiencing common mental health problems	
C: Comparators	Not applicable	Effectiveness/cost-effectiveness studies only: alternative service models, standard care, treatment as usual or inpatient/residential care
O: Outcomes	Not applicable	Effectiveness studies: relevant measures of CYP’s mental health, family functioning, educational attainment or quality of life Cost-effectiveness studies: incremental cost-effectiveness of service model vs. comparator Acceptability studies: qualitative and quantitative data capturing stakeholder views
S: Study design (document type)	Any document containing a sufficiently detailed description of a relevant service	Peer-reviewed empirical studies Effectiveness studies: at least one pre/post relevant outcome measure reported Cost-effectiveness studies: costs, health outcomes or incremental cost-effectiveness analyses reported Acceptability studies: data that directly or indirectly reflected participants’ views of, or participation in, the service reported

For both reviews, two independent assessors extracted data. Disputes were referred to a third reviewer. Quality assessment was conducted for the integrative review only, using the Mixed Methods Appraisal Tool [17].

In the scoping review, descriptions of services for CYP experiencing common mental health problems were mapped to create a service model typology. Synthesis in the integrative review was based on EPPI-Centre methods [18], with the different data sources (effectiveness, cost-effectiveness, acceptability) being analysed separately within each typology group prior to being compared and contrasted across each typology group.

### Primary research phase (case study)

Concurrent with the evidence synthesis phase, service provision across England and Wales for CYP experiencing common mental health problems was mapped using a survey and desk-based methods (e.g., internet searches and follow-up telephone calls). Identified services were coded against the typology so the map could be used as sampling frame for a collective case study [19] of nine services across England and Wales. Services were purposively sampled to capture the spread of different typology models and to reflect characteristics such as service sector (e.g., state-delivered, for-profit, or charitable), locality/setting, target age group and delivery mode.

During this phase, six ‘young co-researchers’ – young adults with lived experience of mental health issues – were employed to work alongside the study’s substantive researchers.

Covid-19 restrictions at the time prevented site visits thus data (except one interview) were collected remotely. Across the nine case study sites, 96 semi-structured telephone/video interviews involving 108 participants (41 CYP, 26 parents, 41 staff) were conducted. The interview schedules (topic guides) for the three participant groups can be found in Additional File 1. Appropriate consent/assent was obtained from all participants. Twenty-two of the 96 interviews were jointly conducted with a young co-researcher. Sites were also asked for information on annual budgets, funding source, and key spending areas.

Case study data analysis was informed by Framework, a matrix-based analytic method widely used in applied health service research [20, 21]. Familiarisation with the data occurred via team members CF, GN, JC, NE, RL, RM and SK reading and discussing the interview transcripts in depth. The transcripts were then coded deductively in NVivo 11 (QSR International, Victoria, Australia) using a thematic framework based on the study’s aims, after which the data were ‘charted’ so that deductive codes for each theme could be examined within each case study site and comparatively across sites. The data were then analysed inductively and iteratively to identify cross-cutting themes, which were shared with the wider research team and the study advisory group. Cost data were descriptively summarised into a table.

### Model building phase

During a series of online and face-to-face meetings, CF, NE, SK and SP firstly compared and contrasted the case study data with the integrative review data using the ‘weaving’ approach to integration through narrative. In the weaving approach, different datasets are analysed, interpreted and reported together on a theme-by-theme or concept-by-concept basis [22]. The three themes emerging from the case study analysis (see below) were used to create three mixed-methods matrices in which the case study data were presented transparently alongside the review data to enable us to look for similarities and differences and see how the case study data might extend understanding of the review data and vice versa.

From these matrices we devised a preliminary model of high-quality services for CYP experiencing common mental health problems that attempted to integrate all the factors associated with access to, navigating, and receiving help from, such services. Using ‘scientific parsimony’ [23], we aimed for an explanatory model of service provision that was comprehensive yet understandable. Importantly, this explanatory model is different to the models in our scoping review typology being technically a ‘meta-model’ or ‘model of models’. However, to avoid overcomplicating things, we simply call it a model.

Our final model evolved through a collaborative process. To make the model usable, accessible and meaningful for commissioners, providers and users of services CF, NE, SK and SP fine-tuned it following feedback from the wider research team, the young co-researchers, the study advisory group, an additional group of young people with lived experience of mental health issues and several CYP’s mental health service commissioners.

## Results

Since the focus of this paper is on model development, results from the evidence syntheses and case study are reported only briefly and for context; results from the model building phase are, however, presented (and subsequently discussed) in detail. Further information on the evidence syntheses and case study can be found elsewhere [24, 25].

### Evidence synthesis

Overall, 310 documents met the inclusion criteria for the scoping or integrative reviews: 296 documents describing 342 services were included in the scoping review. 98 empirical papers were included in the integrative review, 56 providing effectiveness, 62 acceptability, and just three cost-effectiveness data about the various service models (22 papers provided data from more than one perspective). Preferred Reporting Items for Systematic Reviews and Meta-Analyses (PRISMA) 2020 diagrams for the two reviews can be found in Additional File 2.

**Fig. 1:**
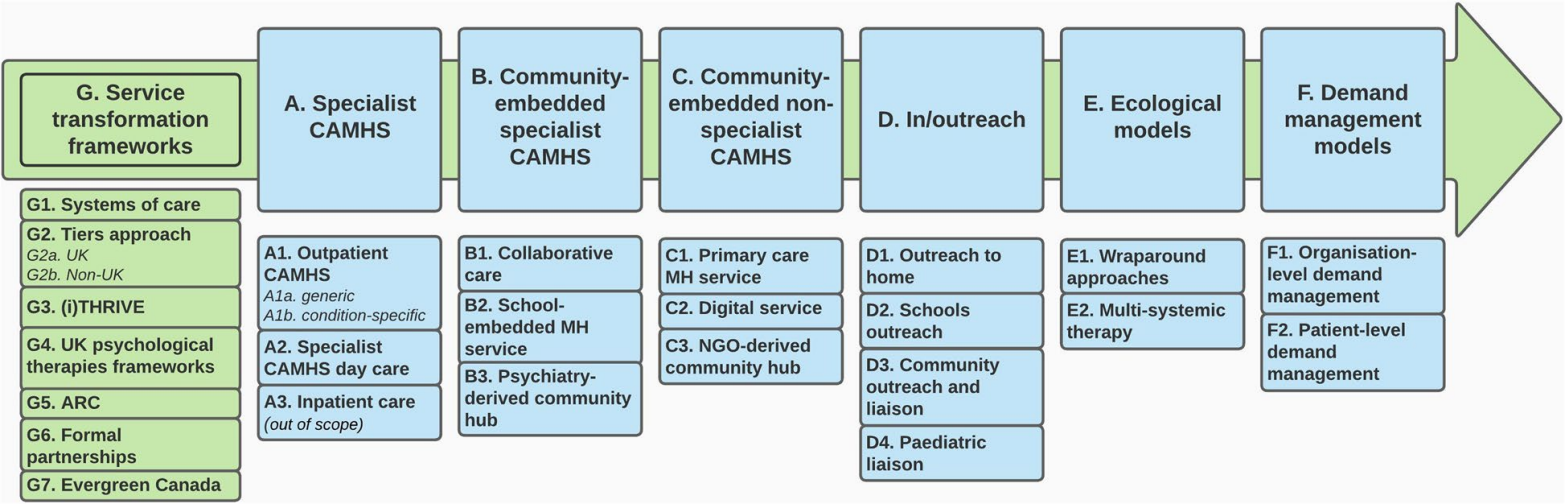
Typology of service models. Note: ARC = Availability, Responsiveness and Continuity; CAMHS = child and adolescent mental health services; MH = mental health NGO = non-governmental organisation; UK = United Kingdom

To simplify the complexities associated with a wide variety of services, the 342 scoping review service descriptions were mapped into a service model typology containing seven broad service model groupings (Fig. 1). For brief descriptions of each model, see Additional File 3.

Across the international literature, the service models most described in the scoping review documents were in/outreach models (Group D), followed by community-embedded specialist CAMHS models (Group B). The latter are models in which formal children’s mental health specialists (e.g., nurses, psychiatrists, psychologists, social workers) work in community settings such as GP surgeries (B1: collaborative care) and schools (B2) rather than hospital settings. Service transformation frameworks (G) were also relatively common.

The integrative review found effectiveness evidence only for collaborative care (B1), outreach approaches in general (D), brief intervention services (a type of patient-level demand management, F2) and the US service transformation framework *Availability, Responsiveness and Continuity* (ARC; G5) [26–28]. The strongest effectiveness evidence was for collaborative care. Cost-effectiveness evidence was very limited (just three papers met the inclusion criteria) with the only robust evidence also being for collaborative care. Since most of the collaborative care evidence was from the US, its applicability to UK health systems is questionable. No service model appeared to be more acceptable to CYP, families or professionals than others.

Our integrative review findings suggest that effective and acceptable services tend to be underpinned by accessibility, interagency working, the use of consultation-liaison and a service culture emphasising child-centredness, user involvement and continuity of care (e.g., seeing the same staff). Brief intervention approaches may be helpful in managing waiting lists since these models often focus on the development of self-management skills.

### Primary research (case study)

One hundred and fifty-four services from 123 different providers across England and Wales were included in the service map. We found service provision across England and Wales to be diverse with statutory (state), private (for-profit) and third (charitable/not-for-profit) sector services operating in a range of settings, supporting CYP with a wide range of common mental health problems. No single model from our typology was particularly dominant. Most services were provided in community, non-health settings, most focused on secondary-school aged children (11–16 years) and most offered support for the most prevalent of the common mental health problems (i.e., general anxiety issues, depression and self-harm). Open access via self- or parent referral was relatively widespread, particularly in the two non-statutory sectors. There was wide variability in the amount of data available, or which sites were willing to provide, on service, training and staff costs. One site provided no cost data at all.

Three themes emerged from the case study data: *pathways to support* (relating to service access and exit); *service engagement*; and *learning and understanding*. Findings of the case study are reported in detail elsewhere [25].

Regarding *pathways to support*, self-referral, the timeliness and availability of support, physical accessibility and planning for support following discharge were important determinants of whether a service is seen as accessible by CYP, families and professionals. A single point of access to services may be beneficial if it does not result in multiple assessments or multiple waiting lists.

The *service engagement* and *learning and understanding* themes highlighted the importance of personalised, holistic and flexible services that involve CYP and families, respect confidentiality, ensure continuity in therapeutic relationships, focus on strengths and engage CYP in creative ways. Staff expertise and professional competence are important but so are empathy and compassion. A positive organisational learning culture (one embracing, for example, continuous professional development, reflective practice, service improvement and involvement opportunities) appears fundamental to service effectiveness and acceptability. Service effectiveness was also linked to opportunities for CYP to develop knowledge and skills that enabled them to both understand and manage their own mental health.

### Model building

A narrative for each of the three themes in the mixed-methods matrices is presented below. Within each theme, we analysed and interpreted the case study and integrative review data across several key concepts, identified in *italics* in each narrative.

1. Pathways to support

*Referral routes* contrasted the advantages of self-referral against those of professional referral. Only case study data were available here; there was no evidence for specific referral routes in the review data. Self-referral was generally seen to promote service access, while professional referral was seen as problematic; poor-quality professional referrals in particular seemed to increase the risk of rejection by a service. In both datasets, there was a consistent perspective on *availability of information* in that lack of information about services made CYP and parents apprehensive and was seen as a barrier to access. Both datasets were also consistent regarding s*peed of access* and *waiting lists*: families generally wanted rapid access to services without lengthy waiting times for both assessment and therapy. The case study data also indicated some services had a role in supporting CYP while they waited for access to more specialist services. *Accessibility at all stages of the CYP’s journey*, picked up in both datasets, reflects frustrations with approaches that nominally improve access but which move bottlenecks further along the CYP’s journey. For example, services with a single point of access may provide more rapid access to assessment but might still have lengthy therapy waits. *Physical accessibility and convenience of the service* was noted in both datasets though in more detail in the case study data. While the integrative review data simply specified that venues need to be accessible, the case study data expands on this by identifying service hours, ability to contact service providers directly and physical convenience (including mode of delivery) as characteristics of accessibility. *Post-service support* was reported in the case study data only and refers to signposting to further support, self-referral back into services, post-discharge follow-up and planning for transition to adult mental health services.

2) Service engagement

There were significant and consistent data supporting the concept of *personalised services* in both datasets, with *involvement* (including service co-design) being central to a personalised approach. CYP and parents want services to be person-centred, age-appropriate, focused on strengths rather than deficits, tailored to individual needs and interests and flexible enough to meet changing needs. In addition, service co-design should be an ongoing process. Furthermore, CYP want services which are engaging, fun and creative. The review and case study data both suggest the third (charitable/not-for-profit) sector may have more freedom and flexibility to offer personalisation than the statutory sector, possibly because third-sector services are rarely diagnosis-led. *Choice* was a key concept in both datasets, though there was more detail in the case study data which reported that CYP and parents want choice in how to access services, the mode of delivery, service setting and the type of support or therapy provided. Moreover, the case study data suggest, where choice is necessarily restricted, it should be clearly communicated to the CYP and family. A significant concept arising in the case study but not the review data was *confidentiality*. The way confidentiality was managed was an important issue for CYP and parents and there were sometimes tensions between confidentiality and safeguarding. Confidentiality can influence decisions about which services CYP will access or the extent to which they will share information with practitioners. For example, CYP may worry about peers finding out about them accessing help in school settings and may therefore prefer at-home, remote telephone or online services. However, remote appointments might not afford the same levels of privacy as office-based appointments. Though identified in both datasets, the case study data provide significantly more detail about *practitioner qualities*, best summarised in the phrase *compassionate and competent staff*. Regarding compassion, staff need to be approachable, non-judgemental, empathic, genuine and passionate about their work with CYP. Regarding competence, CYP and parents want staff to be experienced, knowledgeable and therapeutically skilled; this could mean being seen by an accredited mental health professional rather than a support worker. *Positive relationships* covered therapeutic relationships between practitioners and CYP/families as well as effective team working. Both datasets provided strong evidence for this concept and both included *continuity of care* as important in establishing positive relationships. The case study data identify good communication and practitioner skills as underpinning therapeutic relationships, demonstrating some overlap with *practitioner qualities*.

3) Learning and understanding

The key concept *practitioner learning* was identified in both datasets and refers to staff learning reflectively from other disciplines, from others within their own discipline or, indeed, from the CYP/families in their care. An example from the integrative review is non-mental health specialists gaining knowledge and skills from mental health specialists, particularly in services featuring consultation-liaison. *Acquiring skills for self-care* was also identified in both datasets. The integrative review data outlined that CYP and parents often wanted services to provide them with skills to help themselves. The case study data further expands on this by identifying the nature of these skills: skills to regulate emotions, challenge ways of thinking, manage anxiety and self-soothe, for example. *Personalised approaches to learning* overlaps with *personalised services* discussed in ‘service engagement’ above, with *involvement* being a common characteristic of both. The review data refers to staff who were CYP-centred and the degree to which CYP and parents are involved, while the case study data refers to sessions being pitched at a level that works for individual CYP. *Impact of learning skills for self-care* was substantially more evident in the case study data. Reported impacts in the case study data include reduced anxiety and stress, being better able to cope at school and during the pandemic lockdown, preventing deterioration in mental health, promoting independence, improved resilience and better problem-solving. The review data focused mainly on the impact of brief intervention approaches which are often underpinned by training in self-care skills.

**Fig. 2:**
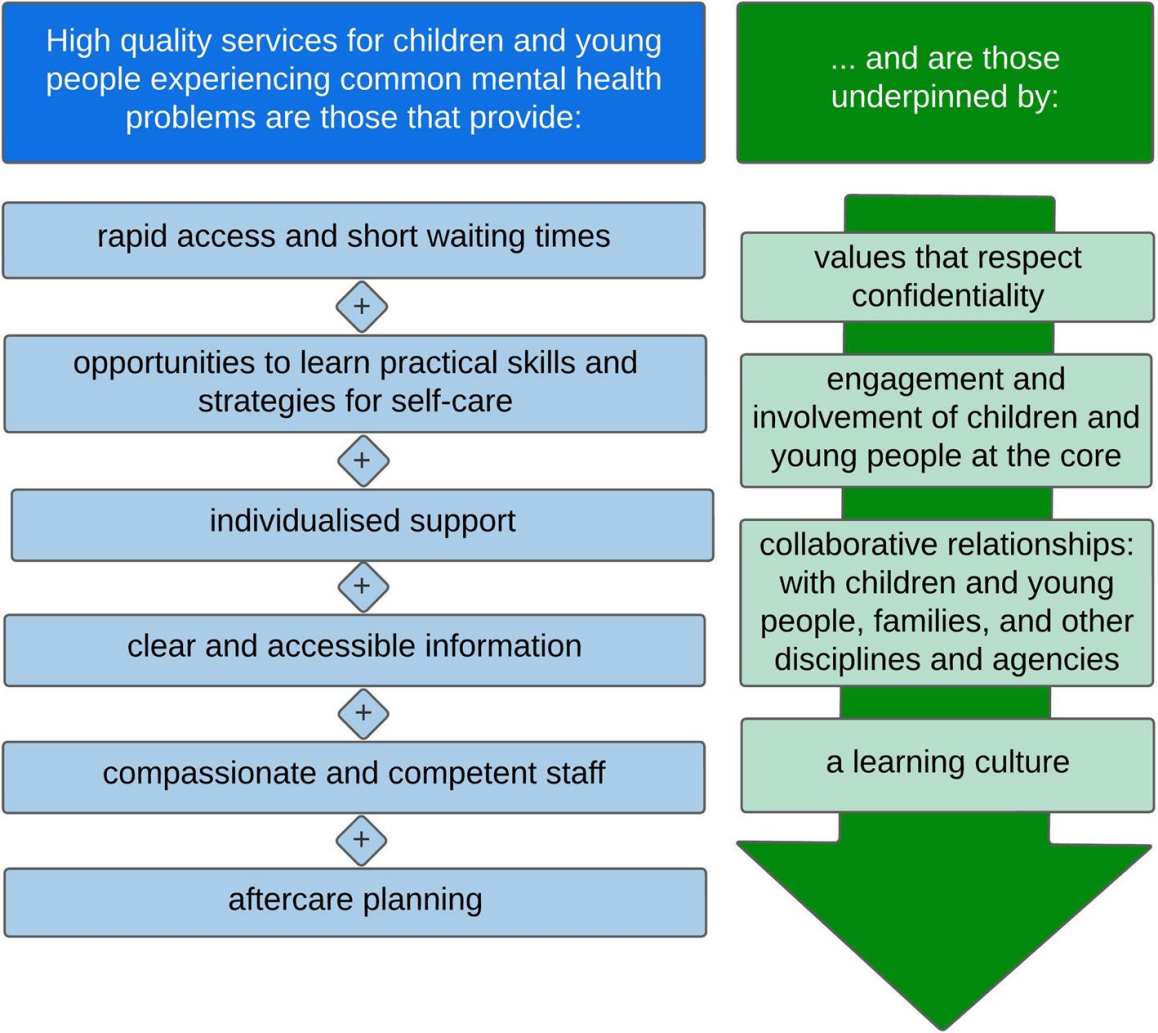
An evidence-based model of high-quality services for CYP experiencing common mental health problems

Integrating the three narratives above, together with feedback from various stakeholders, resulted in a final, general model of high-quality effective and acceptable services for CYP with common mental health problems, represented visually in Fig. 2.

Elements in blue on the left-hand side of Fig. 2 are the core characteristics a service needs to possess to be considered high-quality; green elements on the right are the necessary underpinning characteristics cutting across these core characteristics.

Table 2 further explains the individual model components and outlines how each component was derived from the model building phase’s synthesis (integration through narrative).

**Table 2:** Mapping of themes and key concepts onto the model components

**Model component**	**Explanation**	**Derived from theme (key concepts)**
**CORE CHARACTERISTICS (BLUE ELEMENTS)**		
Rapid access and short waiting times	CYP can access services quickly and, once accessed, therapies and support are provided in a timely manner	Pathways to support (referral routes; speed of access; waiting lists; accessibility at all stages of the CYP’s journey) Service engagement (confidentiality)
Opportunities to learn practical skills and strategies for self-care	The key to providing effective and acceptable support for CYP experiencing common mental health problems is the provision of skills to enable CYP and families to help themselves	Learning and understanding (acquiring skills for self-care; impact of learning skills for self-care)
Individualised support	Services offer choice and flexibility to CYP/families, considering the needs, views interests and hobbies of CYP	Service engagement (personalised services; involvement; choice) Learning and understanding (acquiring skills for self-care; personalised approaches to learning; involvement)
Clear and accessible information	Clear information about services is provided to CYP, parents and professionals using a variety of media (not all families have private access to the internet); information is available to help CYP and families navigate the most appropriate services when given a choice	Pathways to support (referral routes; availability of information)
Compassionate and competent staff	Staff are approachable, caring, empathic and person-centred; staff are appropriately qualified and experienced	Service engagement (practitioner qualities; positive relationships; continuity of care) Learning and understanding (practitioner learning)
Aftercare planning	Processes and systems for navigating out of services as well as into services are available; covers transitions to adult services, ongoing support, potential for re-referrals and continuity of care	Pathways to support (post-service support) Service engagement (positive relationships; continuity of care)
**UNDERPINNING CHARACTERISTICS (GREEN ELEMENTS)**		
Values that respect confidentiality	CYP’s autonomy and perspectives on confidentiality are prioritised and balanced against any safeguarding concerns	Service engagement (confidentiality)
Engagement and involvement of CYP at the core	Activities and therapies provided are accessible, engaging, developmentally appropriate, creative and fun; CYP are involved in shared decision-making for their own care as well as for service design and delivery	Pathways to support (physical accessibility and convenience of service) Service engagement (personalised services; involvement) Learning and understanding (personalised approaches to learning; involvement)
Collaborative relationships: with CYP, families and other disciplines and agencies	Trust is predicated on good interagency/interprofessional relationships and good therapeutic (practitioner-CYP/family) relationships	Pathways to support (referral routes; post-service support) Service engagement (positive relationships) Learning and understanding (practitioner learning)
A learning culture	A learning culture is demonstrated through good team relationships and a reflective learning environment that includes learning from each other as well as from those using services	Service engagement (choice; practitioner qualities; positive relationships) Learning and understanding (practitioner learning)

## Discussion

The main policy driver in England when this study was commissioned (2017) was the *Future in Mind* report [5]. This report outlined a set of key proposals for transforming the design and delivery of children’s mental health services. A government green paper [29] steered a consultation on these proposals during late 2017/early 2018, after which proposals for transforming children’s mental health services were embedded into the five-year *NHS [National Health Service] Mental Health Implementation Plan* (MHIP) [30]. In Wales, the main policy drivers at the time were the all-age *Together for Mental Health* initiative [31] and the NHS-led *Together for Children and Young People* (T4CYP) programme [32]. The 2015 Well-being of Future Generations Act [33] also influenced policy in that it imposed a requirement on public bodies – including health and local authorities – to consider future generations when devising economic, social, environmental and cultural policy.

### Consistency with existing research and policy

The components in our model are consistent with points highlighted in *Future in Mind* and the MHIP, namely that services should offer prompt entry, individualised care, a workforce with the right skills and competencies, welcoming environments, the facilitation of self-care skills and managed transitions to adult services. While the Welsh Well-being of Future Generations Act is a broad piece of legislation and not specific to mental health, two of our necessary underpinning characteristics, collaboration and (public) involvement, are implicit in the Act’s sustainable development principles. More recently in Wales, the NYTH/NEST Framework for Mental Health and Wellbeing (which emerged from the T4CYP programme) identifies six guiding principles for improving CYP’s mental health [34], three of which can be explicitly mapped to our model: trusted adults (values that respect confidentiality); co-produced innovations (engagement and involvement of CYP at the core); and easy access to expertise (rapid access and short waiting times).

Our findings also reflect findings and recommendations from previous UK research and reports, both on children’s mental health services and children’s services in general. More than 15 years ago, a report on young people-friendly general health services [35] identified accessibility, publicity (information), confidentiality, environment, staff attributes (e.g., skills, attitudes and values), joined-up working and CYP’s involvement as markers of quality services. The CAMHS Review [10] noted parents and professionals need information about services, that there should be swifter access to services and that CYP should be able to develop trusting relationships with staff for the length of time they need. Work on self-care support in CYP’s mental health [36] found factors like choice, child-centeredness and staff flexibility to be more important than a service’s theoretical stance or a particular service model. Frith [37] identified co-production with young people, system (multi-agency) working, easily accessible services, workforce development and support for transitions as important factors in children’s mental health services. The National Children’s Bureau [38] stressed the importance of personalising services and therapies for CYP with poor mental health. Finally, Hassan et al. [39] report on a specific service model, Youth Information, Advice and Counselling Services (YIACS; categorised as a C3 model in our typology). Hassan et al. identified opportunities to self-refer, timely provision of support, non-clinical environments, age-appropriate services (personalisation), a non-hierarchical workforce (learning culture) and interagency collaboration as key facilitators of access to, and engagement with, YIACS. These facilitators are strikingly similar to those identified in our model.

### Novel findings

Perhaps the most novel aspect of our findings is that no pre-existing service model, framework or innovation appears to be generally more effective or acceptable than others. Consequently, we argue the focus for those commissioning, designing and delivering services should be on the fundamental components specified in our general model (Fig. 2). As outlined above, some of these components are well-established; others, however, are novel.

Firstly, while support for transitions to adult services is often identified as a service gap, we think *aftercare* is a better concept than transitions since it embraces care continuity, ongoing support post-discharge and re-referral potential – i.e., ‘what happens next?’ – for *all* CYP not just those approaching 18.

Secondly, our model also emphasises the importance of balancing confidentiality against safeguarding and CYP’s autonomy, especially in school-based services. Schools are often seen as ideal places for mental health services [10, 40–42] because they are accessible, closely connected to the community, and relevant staff have daily contact with, and are likely to be trusted by, CYP. Moreover, as non-medical sites, they may be less stigmatising. Yet confidentiality can easily be breached in school settings, for example by reading out lists of students ‘selected’ for a mental health intervention or having counselling/therapy offices located where others might see students attend. In terms of balancing confidentiality against safeguarding, Jenkins [43] notes confidentiality in therapeutic work with CYP, which is often framed in terms of *assumed* legal obligations (e.g., to report abuse or underage sexual activity), may be more nuanced and negotiable than practitioners realise.

A third novel aspect relates to how staff competence and compassion are perceived by those using services. Competence is not just ticking every box in a competency framework; it also covers staff expertise and experience and their capacity to operate within, and recognise the limits of, their own knowledge and abilities. Recognising these limitations fits in with our notion of a learning culture: one embracing reflective and reflexive learning and practice and co-production with CYP and families. It also has implications for peer and associate worker roles since these workers may be best employed *augmenting*, rather than substituting for, trained and experienced mental health professionals. In any case, these workers are likely to benefit from support, supervision and/or mentoring from experienced mental health professionals [44]. There are economic implications too. Attempting to drive down service costs through staff costs (e.g., through self-help apps, associate professionals or peer workers) could be a false economy if supervision and support costs are ignored, if these approaches are assumed to have no impact on service efficacy or if CYP find them unacceptable. Moreover, compassionate care generally requires staff time (or ‘presence’) which may be at loggerheads with the targets and cost savings associated with market-driven health services [45], particularly when, as now, services have significant staff vacancy rates [46].

A fourth novel finding is the importance of a learning culture. While many of the reports cited earlier stress the importance of environment and workforce development, a learning culture also encompasses good team relationships, opportunities for training (at the organisation’s expense) and a reflective learning milieu in which practitioners can learn from each other and, indeed, from service users. Interestingly, some of the most robust effectiveness evidence in the integrative review came from Glisson et al.’s ARC (G5) studies [26–28]. A learning culture is implicit in ARC since ARC sees effective organisations as those demonstrating participation, ‘psychological safety’ (speaking freely without fear of punishment or humiliation), openness to change, responsive rather than reactive services and a commitment to continuous development [26]. *Matrics Plant* [47], a recent all-Wales framework for the development, planning and delivery of ‘psychologically-minded’ services to CYP/families, is also explicit about the value of supervision in facilitating a reflective learning culture.

### Inconsistencies with previous research and policy

Some notionally good practice outlined in *Future in Mind* and the MHIP was not particularly evident in our data and is thus not explicit in our final model. One example is a single point of access. In our data, speed of access and not having long waits for therapeutic support was more important than a single point of access. Rocks et al. [48] found a single point of access has the potential to improve access to children’s mental health services through addressing some of the barriers to access, simplifying where to go to get help and making it easier to contact services. Single access points are, however, not necessarily accompanied by increased capacity and thus do not resolve long waiting times. This reflects the case study data: while a single point of access could facilitate initial access, it could also be confusing to navigate and lead to further assessments and waiting lists for support. The Welsh NYTH/NEST Framework has ‘easy access to expertise’ as one of its core principles; it does not, however, specify how this access should operate.

Another example of notionally good practice is having dedicated (named) staff responsible for mental health in schools. Nothing in our data confirmed this, though this may be sampling artefact. We did, however, identify collaborative interagency relationships as important. While there is ample evidence in the literature that good collaborative relationships between schools and mental health services are important [41, 42, 49, 50], there is a danger that imposing such relationships may not work as well as more organic relationships. In addition, an evaluation of the English ‘mental health services and schools link’ pilots established in response to *Future in Mind* [50] found the resources to implement named contacts were not always available. In Wales, there is a less prescriptive approach with relevant policy containing only broad expectations. For example, whole school approaches are a core principle of the Welsh NYTH/NEST Framework and collaboration between public bodies is a requirement of the Well-being of Future Generations Act.

Given team members’ previous work on self-care support in CYP’s mental health [36], we are not surprised at the inclusion of ‘opportunities to learn practical skills and strategies for self-care’ in our model. In *Future in Mind*, self-care is equated with apps and digital tools, which is a rather narrow view; the Anna Freud Centre [51], conversely, outline an expansive list of activities (such as listening to music, watching television or going outside) that CYP could engage in to help themselves without the involvement of mental health professionals. CYP and parents in the case study sites provided a more nuanced perspective: they certainly wanted the ability to help themselves but wanted services to facilitate this through *supported* self-care (through staff providing them with tangible skills, for example, to help regulate emotions or manage anxiety), often for the period while waiting for more specialist services.

*Future in Mind* and the MHIP expected digital services to play a significant role in future mental health service provision. These expectations were set, however, before the Covid-19 pandemic which provided an external stimulus for the expansion of digital services. That no evidence for digital services met the inclusion criteria for our integrative review is noteworthy though we should add one of our case study sites was a wholly digital service. The general literature on digital approaches is more tempered than the hyperbole seen in some quarters and backs up the notion of digital being an *option* for those who prefer it [52, 53]. A recent review of engagement with digital services in CYP’s mental health [54] found retention rates for digital services were generally good but that the service’s design and modality were important. Risks exist with digital services, however, not present in traditional office-based services (and vice versa). In a recent provider review involving feedback from over 1,700 CYP, the Care Quality Commission [55] concluded that digital services arising out of the pandemic shone a light on health inequalities yet also exacerbated them, and that digital services might miss cues in-person services would not. This latter point was also reported by some service provider participants in our case study. Confidentiality and safety may also be issues. At home, a CYP may be overheard by siblings or parents while using a service, or the CYP may not want parents to know they are accessing services. Moreover, it may be more difficult to offer emergency help remotely, e.g., if a CYP threatens self-harm. A recent rapid review of digital services in CYP’s mental health [52] found digital services were often much briefer than traditional office-based services, leaving no time for identifying action plans or goals.

### Study strengths and limitations

The main study strength – and its principal contribution – is the collaborative development of a comprehensible, evidence-based model of high-quality service design for CYP experiencing common mental health problems that is transferable across services, sectors and geography. We have achieved this via a large and robust study, with a high degree of patient and public involvement (the young co-researchers in particular), entailing an exhaustive evidence review of more than 300 documents and in-depth primary research involving more than 100 stakeholder interviews.

Some service initiatives we were aware of were too new to have filtered through into the literature or service map in any meaningful way, e.g., the introduction in England of schools mental health support teams, alongside a new role of educational mental health worker [29, 30, 56]. The pandemic also brought about a surge in remote/digital (C2) services and while we explored some of these services in our case study sites, these services would have been underrepresented in the literature when our searches were conducted.

We had planned to use observation as a data collection method in our primary research phase; however, Covid-19 restrictions at the time meant we could not directly observe activities at the case study sites and it proved difficult to negotiate observing activities remotely. Thus, we lack the additional insights that observing actions and interactions in a natural setting might generate.

Despite conducting our primary research during the Covid-19 pandemic, we nonetheless recruited to target though we recruited fewer CYP than our sampling goal. However, this should be considered within the context of the consequent lockdowns and school closures. Having to collect data remotely may have both encouraged and discouraged study participation and may have influenced the data generated in interviews. The service provider participant profile was not especially diverse being all White British and 70% female, though this may be a systemic, rather than sampling, issue given 78% of English NHS CAMHS staff are White British and 85% female [46].

We tried to recruit participants who refused or disengaged from services. However, the case study site contacts we asked to facilitate this were unable to recruit such participants. The voices of those who refuse or disengage from services are important and future research would benefit from recruitment approaches (e.g., using social media) that do not rely on the very services they refused to attend or disengaged from.

There was little relevant economic data available in the literature and wide variability in the amount and quality of data available, or which case study sites were willing to provide, on service, training and staff costs.

## Conclusions

Through evidence syntheses and primary research, we have found that a consistent organisational evidence base for service design and delivery in children’s mental health, spanning more than 15 years, appears to have had little impact on service provision. This is particularly salient in the face of post-pandemic rises in demand for such services [57]. We argue that, in the face of this evidence, funders, commissioners and those designing and delivering services for CPY experiencing common mental health problems should focus on the fundamental components necessary for high-quality services rather than impose specific local or national models, frameworks or innovations that are often inflexible and untested. This is a timely argument given the recent establishment of integrated care systems in England, organisations that have the potential to commission and deliver services more creatively and collaboratively. Our evidence-based model can help here: without allegiance to any specific theoretical, philosophical or clinical perspective, it outlines the fundamental components necessary for high-quality services for CPY experiencing common mental health problems, adding significant depth around core issues such as confidentiality, aftercare, personalised approaches, engagement and organisational culture. Moreover, while our model is primarily focused on British service provision, it is broad enough to have utility for international audiences.

We finish with a somewhat ironic research recommendation: since our model is built on, and reflects, a consistent literature spanning many years, we believe there is little value in conducting further exploratory work on the design and delivery of children’s mental health services.

### Supplementary Information

**Additional file 1.** Topic Guides.

**Additional file 2.** PRISMA 2020 Diagrams.

**Additional file 3.** Typology Model Descriptions.

## Data Availability

The datasets generated and/or analysed during the current study are not publicly available due to participant confidentiality but may be available in anonymized form from the corresponding author on reasonable request.

## Abbreviations

ADHD/ADD: Attention-deficit hyperactivity disorder/attention-deficit disorder
ARC: Availability, Responsiveness and Continuity
CAMHS: Child and adolescent mental health services
CAPA: Choice and Partnership Approach
CBT: Cognitive-behavioural therapy
CYP: Children and young people
CYP-IAPT: Children and Young People’s Improving Access to Psychological Therapies (programme)
HSDR: Health and Care Services Delivery Research (programme of the NIHR)
IAPT: (Adult) Improving Access to Psychological Therapies (programme)
IPT: Interpersonal therapy
MH: Mental health
NGO: Non-governmental organisation
NHS: (UK) National Health Service
NIHR: (UK) National Institute for Health and Care Research
PICOS: Population, Intervention, Comparator, Outcomes and Study Design
PRISMA: Preferred Reporting Items for Systematic Reviews and Meta-Analyses
PTSD: Post-traumatic stress disorder
UK: United Kingdom
YIACS: Youth Information, Advice and Counselling Services
