# Supplementary material for: A collaboratively produced model of service design for children and young people with common mental health problems
Source: BMC Health Serv Res. 2024 Jan 24;24:133. doi: 10.1186/s12913-024-10562-7 (PMC10809440; doi:10.1186/s12913-024-10562-7)
Supplement: Supplementary file 1 — Additional file 1. Topic Guides. [file 12913_2024_10562_MOESM1_ESM.docx]

**Additional File 1:** Topic Guides

**1. TOPIC GUIDE FOR CHILDREN AND YOUNG PEOPLE (SERVICE USERS)**

**Context/history**

- Length of time experiencing mental health problems
- Impact of mental health problems
- Who helped/supported you / your family before coming to service?

**Feedback on the service**

- Who told them about the service
- Why were they interested in going/taking part?
- Did they know what to expect? Sufficient info on service provided?
- Pattern / frequency of use of the service
  - Services accessed
  - Who attends
- How easy/difficult is it to access the service?
  - Travel to service (inc. costs)
  - Impact on other activities
- What do they like about it?
- What don’t they like about it?
- Do they feel it has helped them? If so, how?
- Could the service be improved? If so, how?

**Mental health support**

- Have they used other similar services? If so, how does this one compare?
- If other services are involved, who co-ordinates this – is there a key worker or care co-ordinator?
- What would the ideal service look like?
  - How would they like to receive support?
  - Who can best support them?

**Additional questions in response to COVID-19**

I’m interested in finding out if anything has changed recently about the way you get support from [service name] because of Coronavirus:

- Did the way you get support have to change? (e.g., online instead of face to face)?
  - How did you feel about that?
  - What do you like / dislike about these changes?
- How would you prefer to access support in the future?
- What has been the impact of C-19 for you?
- Have your support needs changed in any way as a result of Coronavirus?
  - If new needs have arisen, how might the service best support these?

**2. TOPIC GUIDE FOR PARENTS AND CARERS (OF SERVICE USERS)**

**Context/history**

- Length of time child / young person has experienced mental health problems
- Impact of mental health problems on child and on wider family
- Who helped/supported child and family before the service

**Feedback on the service**

- Who told them about the service
- Why were they interested in going/taking part?
- Did they know what to expect? Sufficient info on service provided?
- Pattern / frequency of use of the service
  - Services accessed
  - Who attends
- How easy/difficult is it to access the service?
  - Encouraging child’s involvement
  - Time to access support (inc. if necessary to take time off work to attend)
  - Costs to travel to service
- What do they like about it?
- What don’t they like about it?
- Do they feel it has helped their child? If so, how?
- Do they feel it has helped them / wider family? If so, how?
- Could the service be improved? If so, how?

**Mental health support**

- Have they used other similar services? If so, how does this one compare?
- If other services are involved, who co-ordinates this – is there a key worker or care co-ordinator?
- What would the ideal service look like?
  - Who can best support their child?
  - How would they like to receive support?

**Additional questions in response to COVID-19**

I’m interested in finding out if anything has changed recently about the way your child/family gets support from [service name] because of Coronavirus:

- Did the way your child/family get support have to change? (e.g., online instead of face to face)?
  - How did you/they feel about that?
  - What did you/they like / dislike about these changes?
- What has been the impact of C-19?
- Have your (family’s/child’s) support needs changed in any way as a result?
  - If new needs have arisen, how might the service best support these?

**3. TOPIC GUIDE FOR SERVICE MANAGERS / COMMISSIONERS**

**Role in the Service**

- current role and length of time at service/in role
- specific training/education in children’s mental health

**Service organisation (N.B., may be outlined in service specification if available)**

- Service aims and theoretical model
- How is the service commissioned and funded?
- Service staffing structure
- Overview of types of services provided and service user profile
- Service referral and access process
  - Eligibility & exclusion criteria (and has this changed over time? Why?)

**View of the service**

- Enablers: What works well and why?
  - Key factors that enable CYP and families to access and navigate service
- Barriers: What works less well and why?
  - Key barriers which impede access and navigation
- Effectiveness: Perception of its impact on children/parents (long and short term)
  - How is service effectiveness measured / evaluated?
    - What happens to this feedback? Does it influence service delivery?
- Perception on the impact of the service on other support services, e.g., primary care, hospital
- Acceptability: Service provider views on what CYP and families like/dislike about the service
  - How is service acceptability measured / evaluated?
    - What happens to this feedback? Does it influence service delivery?
  - What would the ideal service for CYP experiencing common mental health problems look like?
- Service Delivery: How do you ensure the service is viable? Cost effective?
- Is there a consensus that the tasks people do in order to deliver the service are manageable within current workloads? Or, do you think additional staff hours are required? If so, how many hours/in what role?

**Additional questions in response to COVID-19**

I’m interested in exploring service access and delivery and any measures that have been taken in response to COVID-19 at the service:

- Have any services stopped or been paused?
  - What has been the impact of this?
  - Is this change likely to be temporary or permanent?
- Have any services been adapted or delivered differently?
  - Prompt: E.g., rationing; prioritised based on triage; closed to self-referrals?
  - What has been the impact of this?
  - Is this change likely to be temporary or permanent?
- Going forward, do you anticipate any other service changes as result of C-19?
- Prompts: funding; staffing; support needs of CYP/families
