# Supplementary material for: A collaboratively produced model of service design for children and young people with common mental health problems
Source: BMC Health Serv Res. 2024 Jan 24;24:133. doi: 10.1186/s12913-024-10562-7 (PMC10809440; doi:10.1186/s12913-024-10562-7)

**Additional File 2:** PRISMA 2020 Diagrams

Page, M. J., McKenzie, J. E., Bossuyt, P. M., et al. (2021). The PRISMA 2020 statement: An updated guideline for reporting systematic reviews. *BMJ*, n71. https://doi.org/10.1136/bmj.n71

1. **Scoping Review**


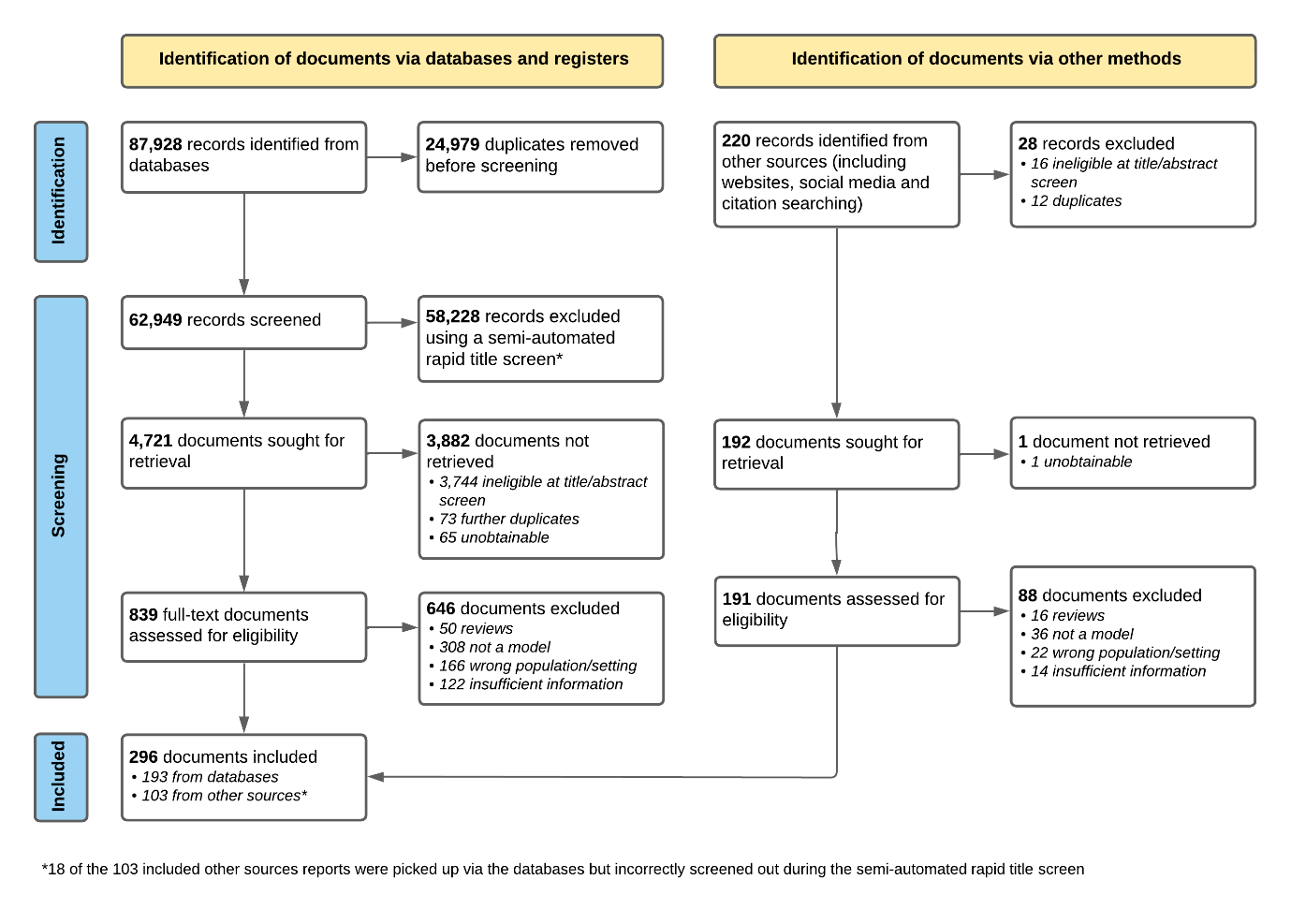


1. **Integrative Review**


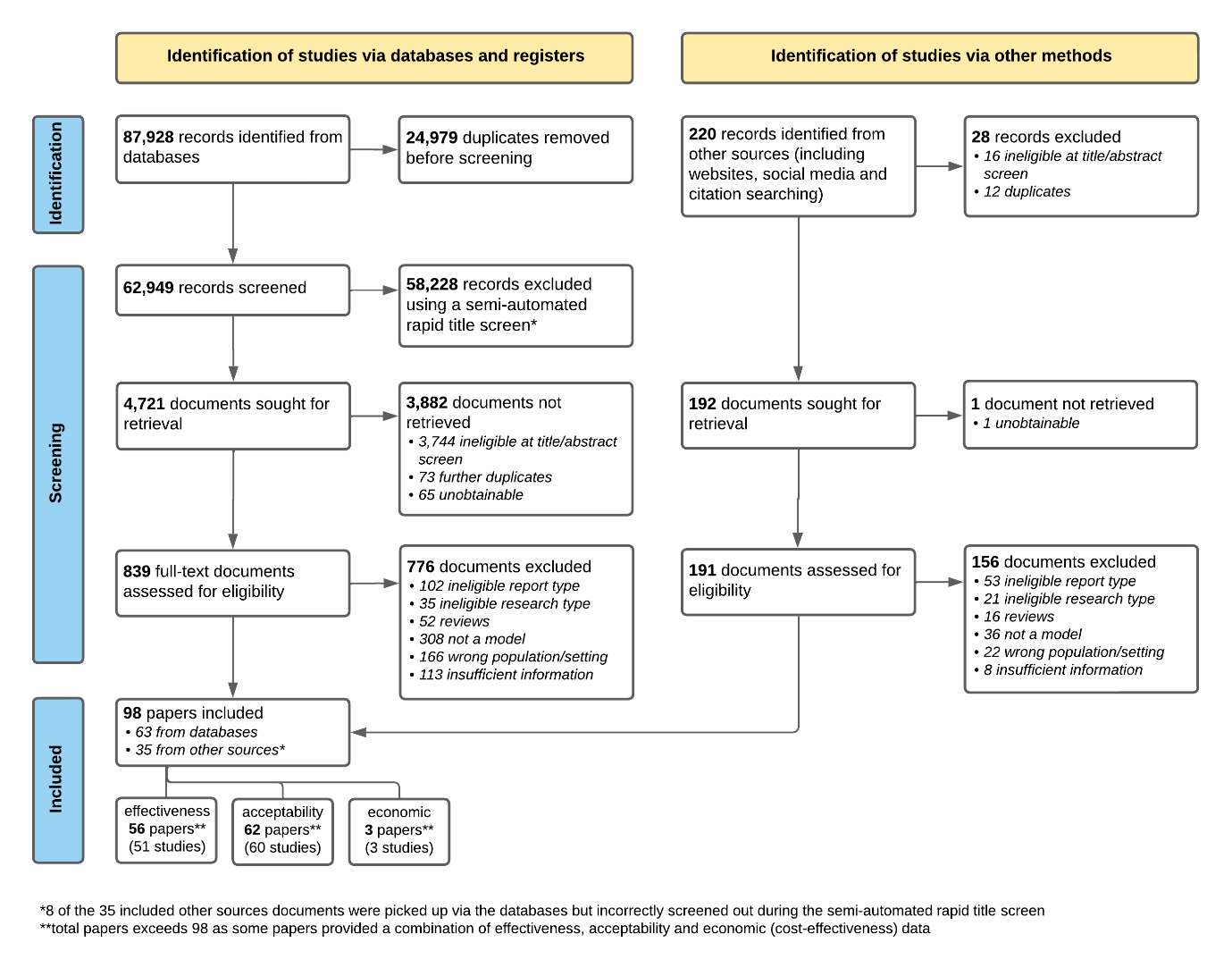

Supplement: Supplementary file 2 — Additional file 2. PRISMA 2020 Diagrams. [file 12913_2024_10562_MOESM2_ESM.docx]
