# Supplementary material for: A collaboratively produced model of service design for children and young people with common mental health problems
Source: BMC Health Serv Res. 2024 Jan 24;24:133. doi: 10.1186/s12913-024-10562-7 (PMC10809440; doi:10.1186/s12913-024-10562-7)
Supplement: Supplementary file 3 — Additional file 3. Typology Model Descriptions. [file 12913_2024_10562_MOESM3_ESM.docx]

**Additional File 3:** Typology Model Descriptions

**Group A: Specialist CAMHS**

The incumbent service model in many countries, encompassing ‘standard’, institutionally based, medically (psychiatry) oriented child and adolescent mental health services (CAMHS). Specialist CAMHS comprises three component services which can operate separately or as a coherent whole: outpatient services (A1); day patient services (A2); and inpatient services (A3). Outpatient CAMHS can be generic (A1a) or targeted at specific conditions (A1b) e.g., ADHD or eating disorders.

**Group B: Community-embedded specialist CAMHS**

Group B and C models are non-hospital or non-institutional services in which key mental health staff are embedded (‘co-located’) within, rather than being ‘visitors’ to, the service. Where key staff visit a service, it is better classified as an in/outreach (Group D) service. What distinguishes B from C models is the key staff in B models are those drawn from traditional Specialist CAMHS (e.g., psychiatrists, psychologists and mental health nurses) whereas C models tend to draw staff from less medically-orientated professions like counselling and youth work.

*B1 collaborative care*

The co-location of specialist CAMHS staff within primary care in order to effect closer and more collaborative working, e.g., a psychiatrist or mental health nurse working in a GP clinic.

*B2 school-embedded mental health service*

These services differ from schools outreach (C3) in that the school employs mental health staff directly or they are embedded into the school for a significant amount of time.

*B3 psychiatry-derived community hubs*

These services have their roots in Australian psychiatrist Patrick McGorry’s work on early intervention in psychosis [1, 2]. B3 models are one-stop (integrated), ‘transdiagnostic’ primary care centres for those aged 12-25 needing help with anything troubling them: mental, physical or sexual health issues; alcohol and drugs; or work and study support. *Headspace Centres* in Australia [3] and *Jigsaw Centres* in Ireland [4] are examples.

**Group C: Community-embedded non-specialist CAMHS**

Similar to B models in being community-embedded, but they differ in that C models tend to have key staff drawn from less psychiatry-orientated professions and access tends to be more open.

*C1 primary care mental health service*

These services are delivered in primary care settings (e.g., GP clinics) but, unlike B1 services, they are not delivered by staff from specialist CAMHS nor are the staff necessarily co-located.

*C2 digital services*

Unlike services which offer telephone or video alternatives to face-to-face contact, C2 services operate solely as digital/remote services. They are normally open access or self-referral services.

*C3 NGO-derived community hubs*

While analogous to B3 services, C3 services have a clear non-medical/non-psychiatry ethos and are usually provided by a non-governmental organisation (NGO). This group includes the UK *YIACS* (Youth Information, Advice and Counselling Services) [5].

**Group D: In/outreach**

Consultation-liaison is the key feature of Group D models.

*D1 outreach to home*

Includes assertive outreach, mobile crisis teams and home treatment. While these services are usually provided for more serious mental health problems, there are some for those CYP at the more complex end of the common mental health problems spectrum.

*D2 schools outreach*

Services that involve mental health experts going into schools to work directly with CYP/families, train and support school staff, and/or carry out preventative and low-level intervention work. They differ from school-embedded (B2) services in that the key staff are ‘visitors’ rather than permanent members of staff.

*D3 community outreach and liaison*

Mental health experts consult-liaise in the wider community (schools and colleges, youth centres, primary care centres and religious buildings, for example). Staff have a wider remit than those working solely in outreach to home (D1) or schools outreach (D2); some D3 services also overlap with paediatric liaison (D4).

*D4 paediatric liaison*

Hospital-based services aimed at improving care through the integration of physical and mental health care in which mental health staff support paediatric (children’s physical health) services through two core business areas: psychiatric emergencies and the supporting the mental health of CYP with (usually long-term) physical health conditions.

**Group E: Ecological models**

Models that consider all aspects of CYP’s lives (school, peers, friends, housing, income, health, wellbeing, criminality and so on) when planning support. Usually underpinned by Bronfenbrenner’s socio-ecological systems theory [6]. Most of these models have their roots in social work or youth offending.

*E1 wraparound approaches*

Wraparound is a collaborative, team-based approach to service and support planning for CYP with complex needs and their families [7]. A service was coded E1 if it made explicit reference to support being ‘wrapped around’ the CYP/family. Wraparound and systems of care (G1; see below) are closely related in that the principles of systems of care are implemented through wraparound.

*E2 multi-systemic therapy (MST)*

MST is a ‘family-ecological’, community-based model targeting young people aged 11-17 at risk of placement in care or custody, devised by psychologist Scott W Henggeler [8]. MST has a rigid set of principles covering assessment, strength-based approaches, multiple systems collaboration and continuous evaluation. MST can be seen as an *individual* level analogue to *system* level wraparound.

**Group F: Demand management models**

Characterised by a need to manage service demand, flow and capacity, often in the face of long waiting lists.

*F1 organisation-level demand management*

In F1 models, an entire service is subject to new ways of working to manage demand, flow and/or capacity. One example is the *Choice and Partnership Approach* (CAPA) [9].

*F2 patient-level demand management*

In F2 services, attempts are made to manage demand by training staff in *brief assessment and intervention* approaches [10]. A limited number of appointments (usually between one and three) are made available for both assessment and therapeutic work.

**Group G: Service transformation frameworks**

Group G frameworks are designed to transform whole systems, often co-existing with the models in groups A to F. Underpinning these service transformation frameworks is an attempt to tackle service fragmentation by encouraging disparate providers to collaborate.

*G1 systems of care*

Systems of care is a US approach to the delivery of CYP’s mental health services [10]. It is a philosophy of care delivery with 13 guiding principles focusing on availability and access, strengths-based individualised services, wraparound (see E1 above), evidence-informed practice, partnerships with CYP/families, integrated services, care (case) management, transitions support and continuous accountability and quality improvement mechanisms. One of the first systems of care was the Fort Bragg initiative.

*G2 tiers approaches*

These are frameworks for organising services according to a hierarchy based on, for example, clinician-assessed need or case complexity that requires some degree of collaboration – or even integration – between service providers. The most well-known tiers approach is the four-tier framework used in the UK [11].

*G3 (i)THRIVE*

THRIVE is a set of principles for creating coherent and resource-efficient communities of mental health devised by the Anna Freud Centre and the Tavistock in London [12]. iTHRIVE is the name for the roll-out (implementation) of THRIVE. Its principal focus is the *needs* of CYP rather than the structures or interventions supposedly meeting those needs. THRIVE identifies five categories of need: ‘THRIVING’; ‘Getting Advice’; ‘Getting Help’; ‘Getting Risk Support’; and ‘Getting More Help’.

*G4 UK psychological therapies frameworks*

Two UK psychological therapies frameworks were picked up in the literature: the *Children and Young People’s Improving Access to Psychological Therapies* initiative (CYP-IAPT) in England and *Matrics Plant* (translated as ‘Children’s Matrix’) in Wales. CYP-IAPT aims to improve the availability of, and access to, evidence-based psychological therapies such as CBT, parenting and interpersonal therapy [13, 14]. Matrics Plant is an all-Wales framework for the development, planning and delivery of ‘psychologically-minded’ services to CYP/families which has similarities to (and notable differences from) CYP-IAPT in England [15].

*G5 ARC (availability, responsiveness and continuity)*

ARC is a US-oriented, focused organisational intervention designed to help community-based mental health services improve their social context and thus address barriers impeding service and treatment outcomes. Its principal proponent is social worker Charles Glisson [16, 17]. ARC has three core assumptions: (1) the application of theories or ideas (i.e., ‘technology’) is a social as well as technical process; (2) mental health services exist in a social context; and (3) service effectiveness is a function of the ‘fit’ between technology and social context. Change agents (drawn from social work, counselling, clinical and occupational psychology) are trained to work at community, organisational and individual levels to bridge identified social and technological gaps.

*G6 formal partnerships*

Three disparate service descriptions – all local and all from England – which appeared to be examples of formal (legal) partnerships between various organisations could not be categorised elsewhere.

*G7 Evergreen Canada*

Evergreen, a Canadian national framework for youth mental health care led by psychiatrist Stan Kutcher [18, 19] could not be categorised elsewhere. It is a framework for Canadian local and federal governments to consider when developing policies, plans and services in CYP’s mental health. Unlike other group G frameworks Evergreen is explicitly non-prescriptive.

**References**

1. McGorry P, Goldstone S. Transforming cultures for the mental health care of young people. In: Hordes M, Gau S, editors. Positive Mental Health, Fighting Stigma and Promoting Resiliency for Children and Adolescents. London: Academic Press; 2016. p. 173–84.

2. McGorry PD, Mei C. Early intervention in youth mental health: progress and future directions. Evid Based Mental Health. 2018;21:182–4.

3. Rickwood D, Paraskakis M, Quin D, Hobbs N, Ryall V, Trethowan J, et al. Australia’s innovation in youth mental health care: The headspace centre model. Early Interv Psychiatry. 2019;13:159–66.

4. O’Keeffe L, O’Reilly A, O’Brien G, Buckley R, Illback R. Description and outcome evaluation of Jigsaw: an emergent Irish mental health early intervention programme for young people. Ir J Psychol Med. 2015;32:71–7.

5. Hassan SM, Worsley J, Nolan L, Fearon N, Ring A, Shelton J, et al. An exploration of young people’s, parent/carers’, and professionals’ experiences of a voluntary sector organisation operating a Youth Information, Advice, and Counselling (YIAC) model in a disadvantaged area. BMC Health Serv Res. 2022;22:383.

6. Bronfenbrenner U. Making Human Beings Human: Bioecological Perspectives on Human Development. Thousand Oaks, CA: Sage; 2005.

7. Bruns EJ, Walker J, Adams J, Miles P, Osher T, Rast J, et al. Ten Principles of the Wraparound Process. Portland, OR: National Wraparound Initiative, Research and Training Center on Family Support and Children’s Mental Health, Portland State University; 2014.

8. Henggeler SW. Multisystemic therapy: an overview of clinical procedures, outcomes, and policy implications. Child Psychol Psychiatr Rev. 1999;4:2–10.

9. York A, Kingsbury S. The Choice and Partnership Approach: A Guide to CAPA. Bournemouth: Caric Press; 2009.

10. Pumariega AJ, Winters NC, Huffine C. The evolution of systems of care for children’s mental health: forty years of community child and adolescent psychiatry. Community Ment Health J. 2003;39:399–425.

11. NHS Health Advisory Service. Child and Adolescent Mental Health Services: Together We Stand. London: HMSO; 1995.

12. Wolpert M, Harris R, Hodges S, Fuggle P, James R, Weiner A, et al. THRIVE Framework for System Change. London: Anna Freud National Centre for Children and Families/Tavistock and Portman NHS Foundation Trust; 2019.

13. Kingsbury S, Rayment B, Fleming I, Thompson P, York A, Hemsley M, et al. CYP IAPT Principles in Child & Adolescent Mental Health Services: Values and Standards: “Delivering with and Delivering Well.” London: CAMHS Press; 2014.

14. Fonagy P, Pugh K, O’Herlihy A. The Children and Young People’s Improving Access to Psychological Therapies (CYP IAPT) Programme in England. In: Skuse D, Bruce H, Dowdney L, editors. Child Psychology and Psychiatry. Chichester: John Wiley & Sons, Ltd; 2017. p. 429–35.

15. Improvement Cymru. Matrics Plant: Guidance on the Delivery of Psychological Interventions for Children and Young People in Wales. Guidance. Cardiff: Improvement Cymru; 2020.

16. Glisson C. The organizational context of children’s mental health services. Clin Child Fam Psychol Rev. 2002;5:233–53.

17. Glisson C, Williams NJ, Hemmelgarn A, Proctor E, Green P. Aligning organizational priorities with ARC to improve youth mental health service outcomes. J Consult Clin Psychol. 2016;84:713–25.

18. Kutcher S, McLuckie A. Evergreen: creating a child and youth mental health framework for Canada. Psychiatr Serv. 2013;64:479–82.

19. Mulvale G, Kutcher S, Randall G, Wakefield P, Longo C, Abelson J, et al. Do national frameworks help in local policy development? Lessons from Yukon about the Evergreen child and youth mental health framework. Can J Commun Ment Health. 2015;34:111–28.
